# Therapeutic Effects of Tai Chi in Patients with Parkinson's Disease

**DOI:** 10.1155/2013/548240

**Published:** 2013-10-31

**Authors:** Hye-Jung Choi, Carol Ewing Garber, Tae-Won Jun, Young-Soo Jin, Sun-Ju Chung, Hyun-Joo Kang

**Affiliations:** ^1^Department of Physical Education, Seoul National University, Gwanak-gu, Seoul 151-742, Republic of Korea; ^2^Columbia University, New York, NY 10027, USA; ^3^Seoul National University, Seoul 151-742, Republic of Korea; ^4^Asan Medical Center, University of Ulsan College of Medicine, Seoul 138-736, Republic of Korea; ^5^Department of Sports Medicine, Soonchunhyang University, 646 Eupnae-ri Shinchang-myeon, Asan-si, Chungnam-do 336-745, Republic of Korea

## Abstract

*Objective*. The purpose of the study was to investigate the effects of a 12-week program of therapeutic Tai Chi on the motor function and physical function of idiopathic Parkinson's disease patients (PDs). *Methods*. The participants were 22 clinically stable PDs in Hoehn-Yahr stages 1-2 randomly assigned to a therapeutic Tai Chi group (TTC, *N* = 11) or a control group (CON, *N* = 9). Two subjects in control group did not complete the study for personal reasons. TTC was performed three days a week (60 min/session). Motor symptoms by the UPDRS were assessed, and tests of physical function were administered before and after the 12-week trial. *Results*. The TTC group, as compared to the CON group, showed changes in the mentation, behavior, mood, and motor scales of the UPDRS (*P* < 0.05, *P* < 0.01, resp.), with no significant main effects on the activities of daily living scale (ADL). However, there was a significant interaction between the time and intervention group on ADL (*P* < 0.05). There were no significant main effects for any of the physical function variables. There were significant interaction effects in balance and agility (*P* < 0.05, resp.). *Conclusions*. This study showed that TTC training had modest positive effects on the functional status of Parkinson's disease patients.

## 1. Introduction

The management of Parkinson's disease (PD) is often enhanced by complementary rehabilitation strategies, such as exercise. For example, studies of resistance [1–3] and endurance [4–7] exercise training have improved balance, gait, postural stability, and physical function and reduced falling in people with PD. Tai Chi, a traditional Chinese martial art that involves meditation and slow, graceful movements, is often recommended to reduce stress, improve mood, flexibility, physical function, and balance [8–10]. Studies of Tai Chi in people with chronic disease including Parkinson's disease and old people have supported the potential for benefit, with gains in the quality of life, postural stability, gait, physical function, immune function, cardiometabolic disease risk factors, and other health-related parameters [4, 11–19]. However, research on the effectiveness of Tai Chi is contradictory due to inconsistencies in the implementation of the Tai Chi movements, limited samples, and the lack of randomized control trials. The purpose of this study is to investigate the effects of a randomized control trial of therapeutic Tai Chi training on improving the motor function and physical function of Parkinson's disease patients.

## 2. Methods

The ethics committee of the Asan Medical Center approved this study. All participants gave informed consent in accordance with the procedures of the Parkinson's Disease Center and the Sports Medicine Center of the Asan Medical Center. 

### 2.1. Participants

The participants of this study were 24 clinically stable patients with diagnosed idiopathic Parkinson's disease recruited from the Parkinson's Disease Center in Asan Medical Center in Seoul, Republic Korea. Eligible participants met the following inclusion criteria: (1) Hoehn-Yahr stage 1 or 2 and (2) stable drug regimen. Volunteers were excluded from the study if they had (1) severe cognitive impairment, (2) concomitant severe neurologic, cardiopulmonary, or orthopedic disorders, (3) specific contraindications to exercise [20], or (4) they had recently participated in any physiotherapy or rehabilitation program. 

### 2.2. Assessment, Randomization, Treatment, and Followup

**Figure 1:**
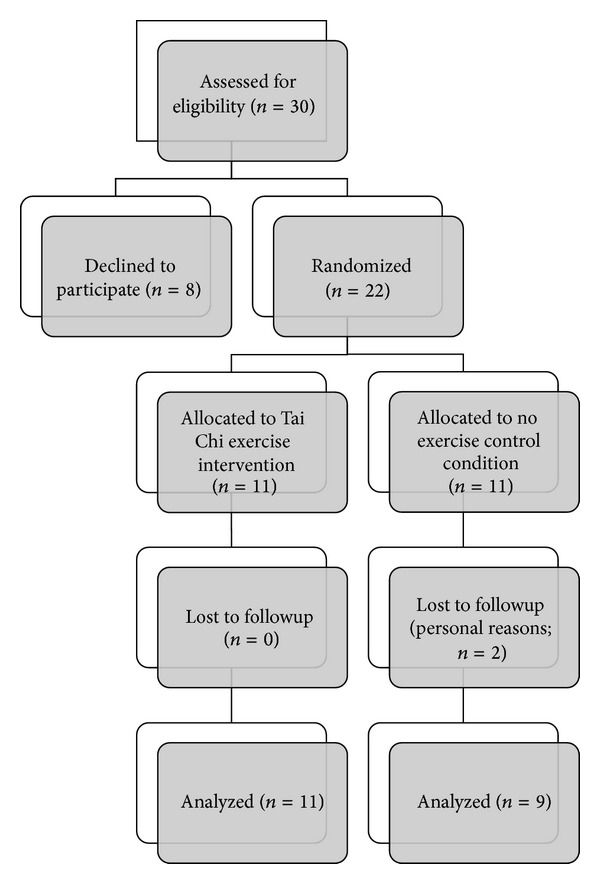
Study flow diagram. Flow diagram of the progress through the phases of a parallel randomized trial of 2 groups of persons with Parkinson's disease who were randomly assigned to a 12-week intervention of therapeutic Tai Chi exercise training or a no exercise control condition.

The volunteers were screened for inclusion and exclusion criteria based upon the medical history and physical examination. Participants were randomized to either a twelve-week intervention of therapeutic Tai Chi (TTC) or a non-exercise control group (see Figure 1). The TTC group visited the clinic 2 times a week and performed home-based activity 1 time per week for 12 weeks. The TTC program is outlined in Table 1. Each TTC session started with a 10-minute stretching exercise warm-up, followed by 30 minutes of Tai Chi exercises, and ending with 10 minutes of meditation and 10 minutes of stretching exercise cool-down. The exercise was performed within the intensity ranges of 11 to 15 (light to somewhat hard) on the Borg ratings of perceived exertion (RPE) scale [20]. Study outcome measures were obtained at baseline (prerandomization; one week prior to the start of the program) and within one week following the end of the 12-week intervention.

### 2.3. Outcome Measures

The outcome measures included several test of physical function (lateral stance, agility, tandem gait, timed up and go, and six-minute walk) and the unified Parkinson's disease rating scale (UPDRS) sections 1–3 [21]. All the tests were performed in the same order, at the same time of day, and when the participant felt best. The evaluator was blinded to the participant's intervention group assignment.

The one-leg standing test is a measurement of balance and functional mobility [22]. The participant stands with hands on hips with eyes closed; when ready, the participant stands on the foot of choice for as long as possible, with the knee of the other leg flexed to 90 degrees. Reaction time was tested by the reaction time to a light signal. The participant stands flat footed on the floor with a monitor screen in front. When a light comes on, they jump up as fast as possible in response to a single light stimulus. The tandem gait test [23] was used to assess balance, appendicular coordination, and gait, which involves multiple sensory and motor systems. The participant walks in a straight line while touching the heel of one foot to the toe of the other with each step. For the timed up and go test [24], the participant was seated in an arm chair (45 cm high) with their back against the chair. On a signal, they stand up, walk 3 meters as quickly and safely as possible, turn around, walk back to the chair, and sit down with their back against the chair. The six-minute walk test [25] was applied as a test of cardiorespiratory endurance for daily physical activities. This test measures the distance that can be walked at a self-paced velocity on a flat hard surface over a period of six minutes. 

### 2.4. Statistical Methods

Descriptive results were expressed as means and standard deviations. An analysis of variance (ANOVA) for repeated measures with one between factor (treatment group; TTC versus control) and one within factor (time; pre- and postintervention) was used to evaluate the effects of the intervention. Significance levels were set *a priori* at *P* ≤ 0.05. All analyses were conducted using SPSS version 15.0. 

## 3. Results

**Figure 2:**
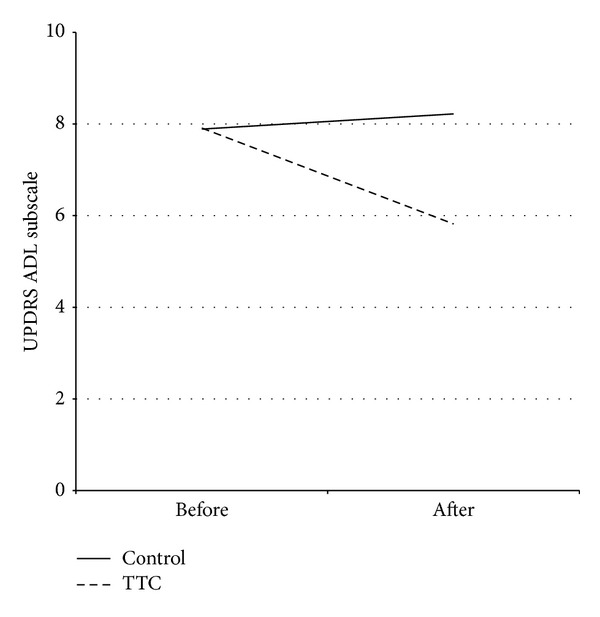
Activities of daily living scores before and after a therapeutic Tai Chi intervention. Mean UPDRS activities of daily living scores before and after a 12-week therapeutic Tai Chi intervention or non-exercise condition in persons with Parkinson's disease. The results of the ANOVA showed a significant interaction effect (*P* = 0.037). Abbreviations: TTC, therapeutic Tai Chi.

The study participant flow is shown in Figure 1. A total of 11 participants were randomized to the TTC and 11 participants to the control condition. Two subjects in the control group did not complete the study for personal reasons unrelated to the study.  Table 2 presents the characteristics of the participants who completed the study. As shown in Table 3, significant changes were observed in the mentation, behavior, and mood subscale and the motor subscale of the UPDRS, with no significant main effects on the activities of daily living scale (ADL), balance, or reaction time. However, there were significant interactions between time and intervention group on the UPDRS activities of daily living subscale (Figure 2). No significant effects were observed for any other measures.

**Figure 3:**
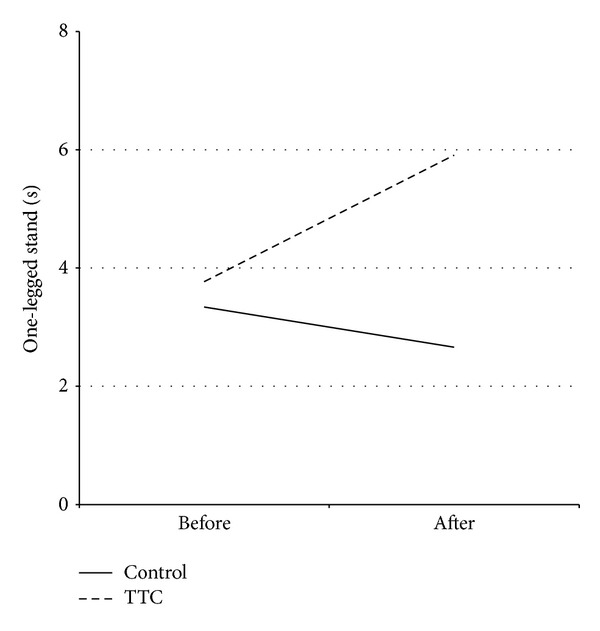
Balance before and after a therapeutic Tai Chi intervention. Mean one-leg balance time (one-leg standing test) before and after a 12-week therapeutic Tai Chi intervention or non-exercise condition in persons with Parkinson's disease. The results of the ANOVA showed a significant interaction effect (*P* = 0.035). Abbreviations: TTC, therapeutic Tai Chi.

**Figure 4:**
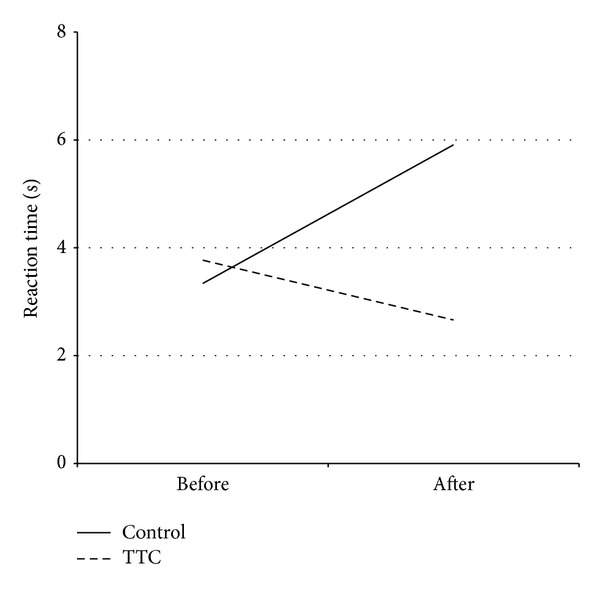
Reaction time before and after a therapeutic Tai Chi intervention. Mean reaction time (light stimulus test) before and after a 12-week therapeutic Tai Chi intervention or non-exercise condition in persons with Parkinson's disease. The results of the ANOVA showed a significant interaction effect (*P* = 0.035). Abbreviations: TTC, therapeutic Tai Chi.

Functional measures before and following 12-weeks of TTC are shown in Table 4. There were no significant main effects for any of the variables. However, as shown in Figures 3 and 4, there were significant (*P* < 0.05) interaction effects for balance (one-leg standing test) and reaction time (light stimulus).

## 4. Discussion

In this study, we investigated the motor and nonmotor effects of therapeutic Tai Chi exercise training on participants with PD. The findings of our study showed modest effectiveness of Tai Chi training on people with Parkinson's disease, with significant interaction effects for balance, reaction time, and ADLs. These findings support that Tai Chi is effective in improving some aspects of motor function, most notably reaction time and balance. In addition, PD participants reported an improvement in their ability to engage in their activities of daily living.

Exercise therapy has a major role in the rehabilitation of the patients with Parkinson's disease [26, 27]. Regular physical activity may increase the functional ability and enhance the capacity for independent living by decreasing the need for assistance with the activities of daily life [27]. In addition, exercise may achieve the goal of neuroprotection, slow disease progression, and postpone disabilities [28, 29].

Several cohort studies have described the functional achievements of moderately affected Parkinson's disease participants who have undergone intensive standardized exercise training [30–33]. However, Burini et al. [4] reported in a study with a crossover design that a 7-week Tai-Chi and aerobic exercise training did not affect the severity of neurological signs and symptoms as assessed by the UPDRS or have any significant impact of training on the attendant disability. Review papers by Kwakkel et al. [34] and de Goede et al. [35] showed that physical therapy is mainly focused on mobility rather than various neurologic symptoms such as rigidity and tremor. Brusse et al. [36] reported that some aspects of the mobility and balance functions were positively affected by Tai Chi exercise. However, assessing the mobility and balance function by only one item, such as the walking function, is not very representative of the spectrum of the motor signs and symptoms. Furthermore, there is little attention toward non-motor symptoms such as fatigue mood and health quality of life, all of which may be altered by exercise [27, 37–40]. In a recently published clinical trial, Li et al. [31] found beneficial effects of Tai Chi exercise on balance, physical function, and falls, suggesting that Tai Chi is an appropriate physical activity for patients with Parkinson's disease and it might be useful as a therapeutic exercise.

The ability to balance is related to the control of the center of gravity within the base of support. Many persons with PD report impaired balance and falls [11, 41]. Koller and Huber [42] found that the balance impairment in older adults with a longer duration of PD does not usually respond to levodopa; 38% of the persons with PD experience fall, 13% fall more than once per week, and some studies have reported that PD patients fall repeatedly throughout the day. Persons with PD are 5 times more likely to suffer falls-related injuries such as hip fractures than healthy older adults. 

Reaction time for agility was measured in the present study and improved with TTC training. TTC training has benefits for postural stability in elderly people, including patients with PD, likely by acting on a number of sensorimotor systems that contribute to postural control [12, 43, 44]. This improvement combined with the observed improved agility performance confirms the separate observations of previous studies showing that TTC training results in better balance capacity, proprioception function, and muscle strength [11, 45–47]. While our study and other studies have shown enhanced balance and agility resulting from Tai Chi, further work is needed to confirm that these improvements in fall risk factors actually result in fewer falls, as there are somewhat contradictory findings in the literature [19, 45, 48].

Physical function refers to the assessment of the capability to complete a specific task rather than assessing the physiologic-derived attributes, and it is believed to reflect the ability to carry out activities of daily living [39]. In the present study, we assessed the physical function with the timed up and go, tandem gait, and six-minute walk tests. The timed up and go test showed no significant change after training, which is in contrast to the findings of Li et al. [31]. These differences may be due to differences in the programs of Tai Chi, the wider range of disease severity in the study by Li et al., or the smaller numbers of participants in our study. The timed up and go test largely depends on the muscular power and strength of the quadriceps and the hip extensor muscles to arise from a chair [49, 50]. It may be that our version of Tai Chi does not substantially improve lower extremity power. The results of the tandem gait test and the six-minute walk test were also not changed after 12 weeks of TTC training. These results indicate the significant relationship between cardiorespiratory fitness and functional fitness. Poor performance on the tandem gait and six-minute walk could result from several potential factors, including poor cardiorespiratory fitness and gait or balance abnormalities [23, 51]. Many of the PD participants likely had poor cardiorespiratory fitness and inadequate levels of physical activity, as shown by Garber and Friedman [39]. Consistent with this finding, all of the participants in this study had below normal results on the six-minute walk test, suggesting that poor cardiorespiratory fitness, gait, and/or balance may have been an important determinant of performance [39]. Other studies of Tai Chi have not shown an improvement in cardiorespiratory fitness [52], and our results are consistent with these previous findings.

This study investigated motor function and the severity of motor and non-motor symptoms and signs. These motor and nonmotor symptoms and signs were not improved after treatment, although self-reported engagement in activities of daily living was enhanced by Tai Chi exercise. TTC training involves splitting up complex movements into simple motor tasks and incorporating simultaneous movements, which is also beneficial for Parkinson disease patients. Secondly, physical functions, namely, balance and agility, were improved. Thus, this study lends additional evidence for Tai Chi as a complementary treatment for people with Parkinson's disease—one that will allow them to engage more fully in their activities of daily living. Further studies are needed that will consider the effects of various components of a Tai Chi program and also to help to identify the intensity, duration, and frequency of Tai Chi exercise to attain optimal benefits. Further, the influence of various medication and dietary factors may moderate the effects of exercise, and this has also not been studied. 

## Figures and Tables

**Table 1:** Weekly content of the Tai Chi exercise program.

Week	Tai Chi exercise
Week 1	Orientation, basic stretching

Week 2	Abdominal breathing, neck and shoulder movement exercise

Week 3	Abdominal breathing, trunk, hip, knee, and ankle movement exercise

Week 4	Abdominal breathing, summary of TTC warm up, relaxation

Week 5	Abdominal breathing T1. Commencing movement T2. Opening and closing hands T3. Single whip

Week 6	Abdominal breathing T4. Waving hands in the cloud T5. Closing movement

Week 7	Abdominal breathing Review and practice

Week 8	Abdominal breathing T6. Brush knee T7. Playing lute

Week 9	Abdominal breathing T8. Perry and punch

Week 10	Abdominal breathing T9. Block T10. Opening and closing

Week 11	Review and practice

Week 12	Review and practice

**Table 2:** Characteristics of 20 participants with clinically stable mild to moderate Parkinson's disease randomized to therapeutic Tai Chi or a no exercise control condition.

Variable	Treatment group	
	Tai Chi	No exercise control
Number (*n*)	11	9
Age (years)	60.81 ± 7.6	65.54 ± 6.8
BMI (kg·m^−2^)	24.93 ± 3.7	25.41 ± 3.0
Years since diagnosis	5.2 ± 2.7	5.2 ± 2.7
Hoehn-Yahr stage	1.6 ± 0.6	1.8 ± 0.3

Note: table values are means ± standard deviations.

There were no significant differences between groups on any of the variables.

**Table 3:** Unified Parkinson's disease rating scale (UPDRS) subscale scores before and after 12 weeks of Tai Chi training or a no exercise control condition in 20 participants with mild to moderate Parkinson's disease.

UPDRS subscale	Treatment group	Time		*P*=		
		Before	After	(*a*)*	(*b*)^†^	(*a* × *b*)^‡^
Mentation, behavior, mood	Tai Chi	2.18 ± 2.13	1.27 ± 1.84	0.025	0.947	0.411
	Control	2.0 ± 1.58	1.56 ± 1.33			

Activities of daily living	Tai Chi	7.91 ± 1.81	5.82 ± 3.37	0.119	0.378	0.037
	Control	7.89 ± 3.62	8.22 ± 3.70			

Motor scale	Tai Chi	22.36 ± 7.44	15.64 ± 9.73	0.010	0.600	0.062
	Control	17.67 ± 8.21	16.44 ± 9.08			

Note: table values are means ± standard deviations.

*(*a*): main effects for time (pre- versus postintervention).

^†^(*b*): main effects for intervention treatment group (Tai Chi versus control).

^‡^(*a* × *b*): time × group interaction.

**Table 4:** Tests of physical function before and after 12 weeks of Tai Chi training or no exercise control condition in 20 participants with mild to moderate Parkinson's disease.

Physical function test	Treatment group	Time		*P*=		
		Before	After	(*a*)*	(*b*)^†^	(*a* × *b*)^‡^
One-leg standing (seconds)	Tai Chi	3.34 ± 3.01	5.91 ± 4.22	0.378	0.229	0.035
	Control	3.77 ± 2.43	2.66 ± 1.89			

Reaction time (seconds)	Tai Chi	489.8 ± 140.2	467.1 ± 102.1	0.085	0.819	0.016
	Control	432.1 ± 133.7	554.4 ± 228.7			

Timed up and go (seconds)	Tai Chi	7.39 ± 0.89	7.03 ± 0.90	0.294	0.173	0.066
	Control	8.06 ± 2.91	9.32 ± 4.16			

Tandem gait (seconds)	Tai Chi	8.77 ± 2.15	8.55 ± 1.34	0.985	0.146	0.801
	Control	10.10 ± 5.05	10.35 ± 2.88			

Six-minute walk (meters)	Tai Chi	442.6 ± 71.7	472.1 ± 58.6	0.284	0.079	0.241
	Control	369.9±139.7	368.6±152.6			

Note: table values are means ± standard deviations.

*(*a*): main effects for time (pre- versus postintervention).

^†^(*b*): main effects for intervention group (Tai Chi versus control).

^‡^(*a* × *b*): time × group interaction.
